# Importance of Cardiac Rehabilitation and Mouth Opening Exercises in Oral Squamous Cell Carcinoma: A Case Report

**DOI:** 10.7759/cureus.50954

**Published:** 2023-12-22

**Authors:** Sarang S Bhoyar, H V Sharath, Shraddha S Kochar, Sharvil Nerkar

**Affiliations:** 1 Paediatric Physiotherapy, Ravi Nair Physiotherapy College, Datta Meghe Institute of Higher Education and Research, Wardha, IND

**Keywords:** cardiac rehabilitation, anterolateral thigh flap, pain, physical therapy, oral squamous cell carcinoma

## Abstract

The most prevalent malignant tumor in the oral cavity is squamous cell carcinoma (SCC). Social interactions are impeded, including eating, conversing, and practicing basic oral hygiene. A 43-year-old man who had previously suffered pus discharge in the right side of the buccal mucosa complained of dull aching in the lower right back of his jaw. The patient's right buccal mucosa was surgically treated for SCC two years ago. He received 30 cycles of high-dose radiation therapy for SCC of the right buccal mucosa. The right maxilla and mandible had osteoradionecrosis, according to a clinical and radiographic assessment. The aim of emphasizing the importance of cardiac rehabilitation and mouth opening exercises in oral squamous cell carcinoma (OSCC) is to address the multifaceted impact of this type of cancer on a patient's overall health and well-being. OSCC refers to a type of cancer that occurs in the cells lining the oral cavity, including the lips, tongue, gums, and the floor of the mouth. The implications of OSCC go beyond the local effects on the oral region and can have systemic consequences, affecting various aspects of a patient's health.

## Introduction

The oral cavity is commonly considered to be the most prevalent location for carcinoma, depending primarily on geographic regions as well as gender of the patient. India has a high rate of oral cancer due to the country's widespread use of smoking and chewing tobacco [1]. Oral cancer is also more common in people who smoke, have poor eating habits, have less education, live in remote areas, have a sedentary lifestyle, and have difficulties maintaining dental and oral hygiene. It is described as a tonic contraction of the masticatory muscles, causing a progressive shutting of the mouth. The effects of this disorder include malnutrition, poor oral hygiene, slurred speech and eating, and difficulties seeking dental care [2]. Malignant lesions affecting the cervical lymph nodes account for more than 50% of cases [3,4]. The complications of head and neck cancer (HNC) radiation therapy include osteoradionecrosis (ORN) of the jaw [5]. In addition to complementary therapies such as head and neck exercises, mouth opening drills using therapeutic tools, and mouth proprioceptive neuromuscular facilitation (PNF) technique, physical therapy may be beneficial for patients with oral cancer [6]. Regaining a functional range of motion (ROM) is the primary objective of a postoperative patient's physiotherapy treatment plan since it will improve their quality of life (QOL) [7]. A few examples are mouth PNF exercises, breathing drills, mouth opening exercises, assistive device drills, bilateral upper and lower limbs mobility exercises, and neck mobility exercises [8,9]. Impairment in swallowing is a major adverse effect of chemotherapy [10,11].

The adverse effect of HNC treatment may be an immediate one that goes away after treatment, or it may be a lingering one [12]. Behavioral therapy, food modifications (bolus size and texture), and swallowing exercises can all be used to alleviate dysphagia-related problems. Shoulder dysfunction is a common complication of HNC treatment [8]. Radiation and neck dissections can also cause damage to the shoulder joint [13]. Limited mouth opening, commonly known as trismus or jaw hypomobility, is a common symptom of cancer patients. Patients who have restricted mouth opening experience detrimental effects on their health and QOL [14,15]. Oral access to dental care and oncologic surveillance may also be limited to a varying degree. Restricting mouth opening makes it very difficult to maintain an open airway and considerably increases the risk of aspiration [16]. Inactivity eventually results in muscles atrophying and shrinking, and joint surfaces deteriorating, which limits the ROM. Esophageal, pharyngeal, and oral phases make up a normal swallowing function. The structure and physiology of the mouth cavity enable the tongue to drive food and fluids into the throat during the oral phase of swallowing [17].

It is believed that the mandibulae's decreased mobility and ROM are adaptive mechanisms intended to halt further damage and hasten recovery when there is pain. At the time of diagnosis, up to 85% of people with HNC report having mouth pain. As changes in mouth opening varied greatly, no stretching technique was found to be superior to others in either preventing or curing trismus [14]. Adjuvant analgesics, psychological counselling, physical therapy, and invasive treatments such as the injection of medications into the spinal column, neural blockade, and reprobative approaches are some other options for pain management [14]. Chemotherapy with three weeks of 100 mg/m2 cisplatin is used to treat postoperative high-risk, locally advanced squamous cell carcinoma of the head and neck [18]. Transfer of glucose and other hexose substances, such as fructose, is mostly carried out by the glucose transporter (GLUT) family of transporters, which is composed of transmembrane proteins produced by solute carrier family 2 member 1 (*SLC2A*) genes [16].

## Case presentation

Patient information

A 43-year-old man who had been complaining of pain and pus discharge from the lower right back of his jaw for about six months presented to Acharya Vinoba Bhave Rural Hospital (AVBRH), Wardha, Maharashtra, India. The patient stated that until he saw growth on the right back portion of his cheek six months ago, he seemed to be in good health. The outgrowth started gradually and expanded to its current size of approximately 4x3 cm. The pain was gradual in onset, dull aching, intermittent, progressive, worsened on chewing, and eventually subsided on its own. Furthermore, he revealed a history of chewing gutkha and tobacco four to five times each day for almost 30 years. The patient presented to AVBRH with complaints of pain and pus discharge from the lower back of his jaw for about six months, and also he was having an outgrowth. To rule out the diagnosis, investigation included a contrast-enhanced computed tomography (CECT) of the buccal cavity. The patient was diagnosed with oral squamous cell carcinoma, and, consequently, was advised to undergo fine needle aspiration cytology for a suspicious soft tissue area located near the right mandibular premolars and in front of the fatty tissue of the flap. According to the Numerical Pain Rating Scale (NPRS), the pain was 6/10 in severity. Avoiding strenuous activity of mouth activities helped relieve jaw pain, which increased when the mouth was opened or during the chewing process. Due to all of these concerns, the patient was referred to the physiotherapy rehabilitation center. The patient's main concerns were trouble opening his mouth after surgery, difficulty in speaking, difficulty in performing activities of daily living, and reduced lung lobe secretion.

Clinical findings

The patient's written and verbal consent was obtained. On observation, the patient was seen lying in a supine position with the head supported with a pillow underneath, both elbows and shoulders in a neutral position, and hip and knee extended. A tracheostomy tube was present. As part of a neurological assessment, a sensory examination was performed, and all sensations (temperature, pinprick, and light touch) were intact. The patient was well-oriented to time, person, and place. The patient's vital signs included a pulse rate of 76 beats per minute, respiratory rate of 16 breaths per minute, and a blood pressure of 130/90 mmHg. The temporomandibular joint’s (TMJ) (mouth opening) ROM before physical treatment was two fingers. The right infraorbital area and maxilla were both tender, with grade 2. The left submandibular lymph node was firm and non-tender to the touch.

Radiological findings

**Figure 1:**
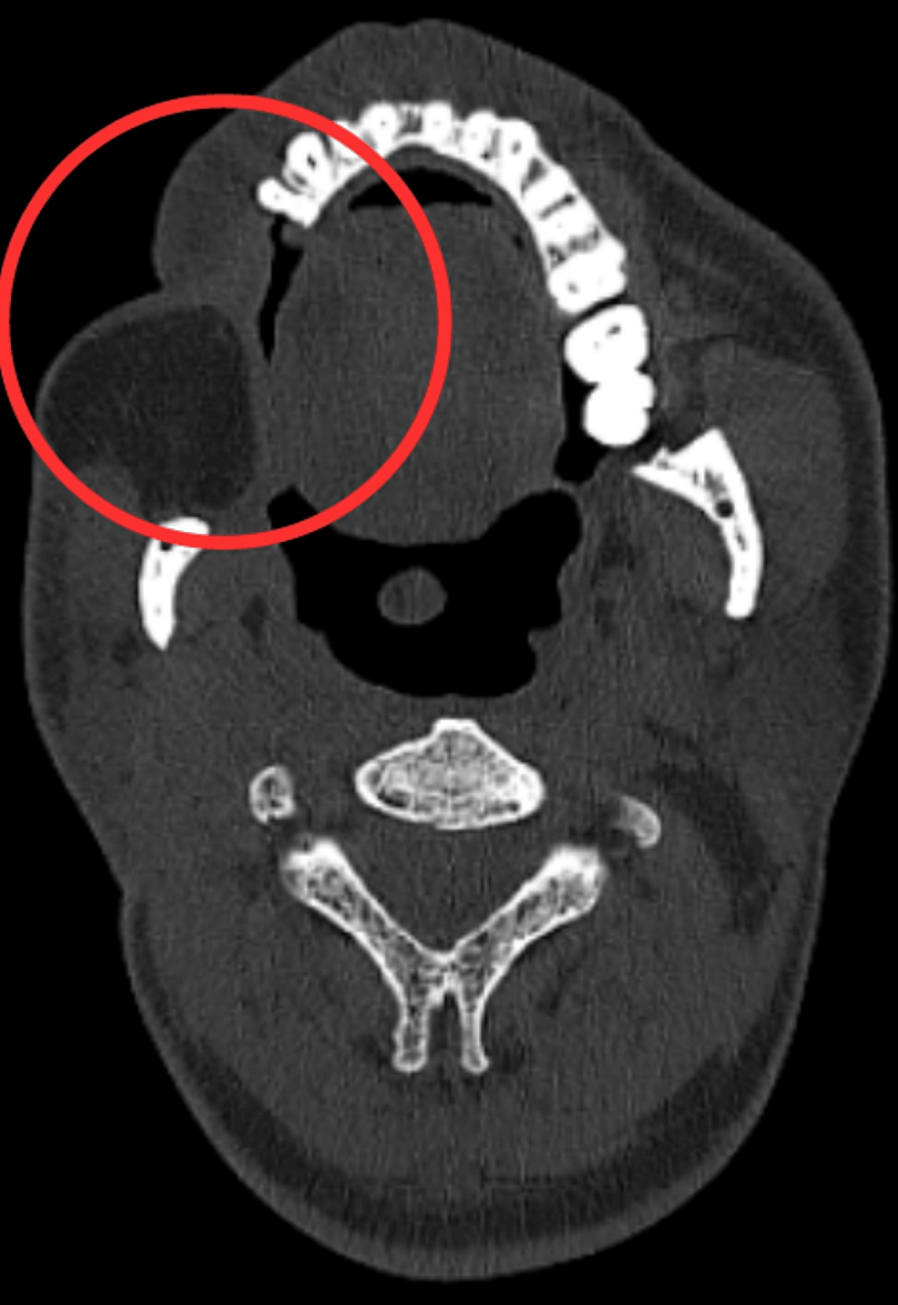
CECT of the buccal cavity CECT, contrast-enhanced computed tomography

Figure 1 shows preoperative CECT. The size and extent of the original mass lesion were determined through a CECT. A large heterogeneous lobulated enhancing mass at the buccal space on the right aspect was seen. There was no apparent fat plane connecting this mass to the right masseter muscle, and it continued posteriorly toward the right masticator area.

Physiotherapy intervention

Week 1

**Figure 2:**
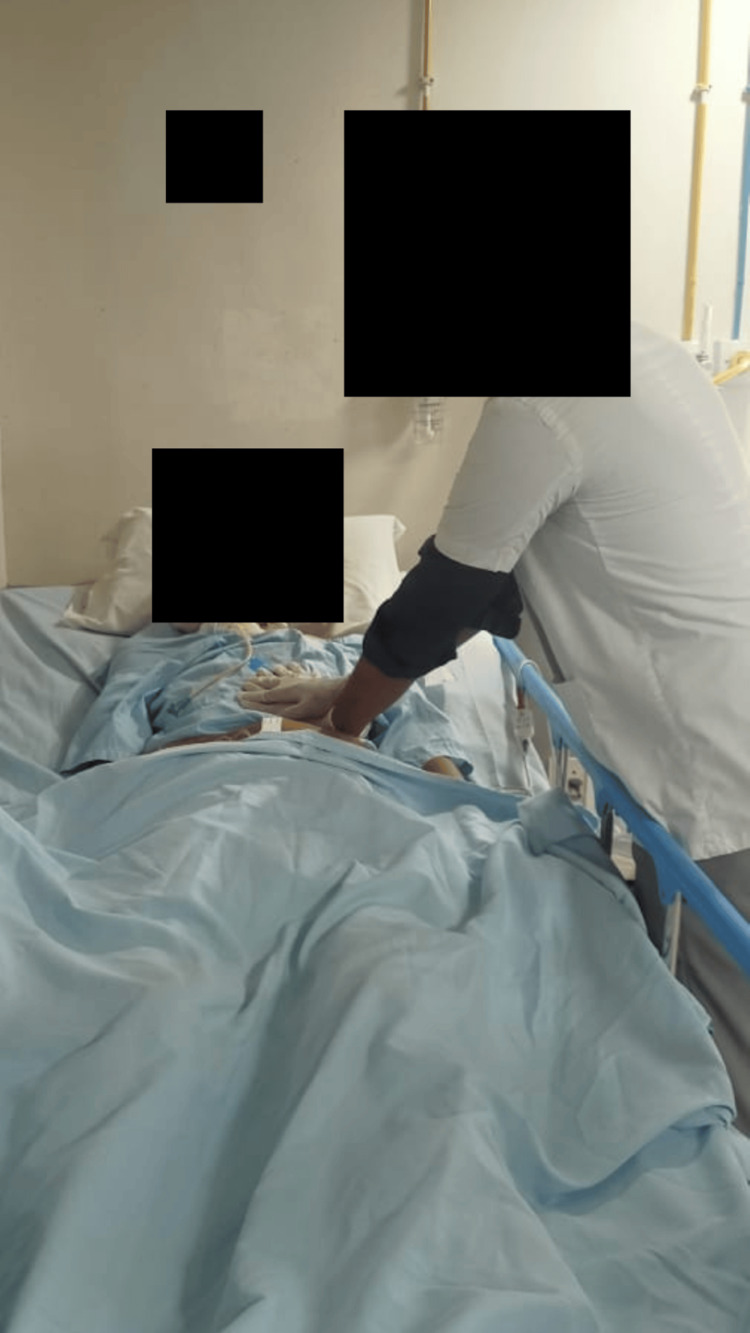
Manual chest vibration given on the left lower zone

In addition to tongue protrusion, exercises for opening and closing the mouth were taught. Simple passive upper and lower extremity exercises were taught to improve and maintain circulation. Vibration and chest percussion were used to remove pulmonary secretion (Figure 2).

Week 2

Exercises such as 4x4 (four exercises, four times), which involved useful jaw opening exercises, maintain ROM lateral deviation, lateral deviation, and available mouth opening, and help enhance the ROM of the TMJ.

Weeks 4-6

**Figure 3:**
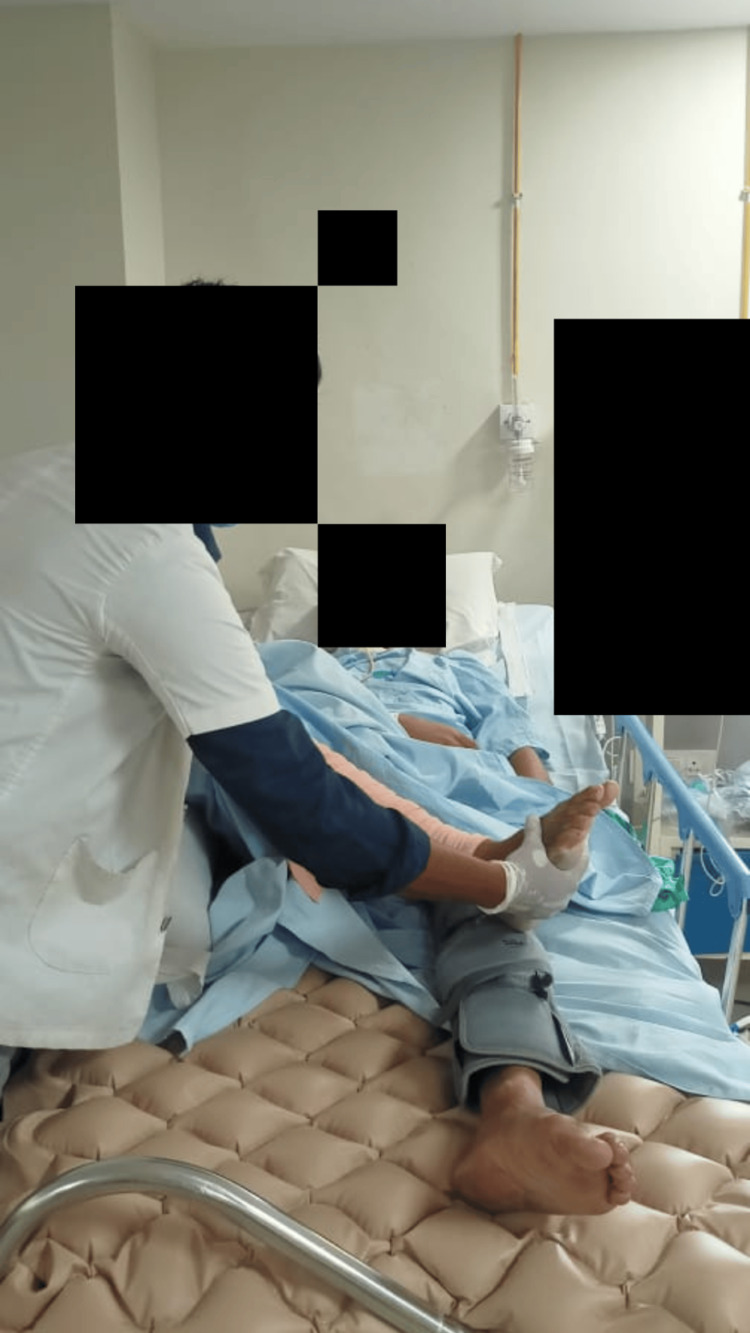
D1 flexion and extension pattern of the PNF PNF, proprioceptive neuromuscular facilitation

Exercises involved keeping the mouth open for 10 seconds while opening and closing it. Stretching with PNF improved ROM and was carried out once or twice a week. D1 flexion and extension pattern of the PNF technique was used to improve the ROM (Figure 3). Table 1 shows the physiotherapy protocol.

**Table 1:** Physiotherapy intervention ROM, range of motion; TMJ, temporomandibular joint; PNF, proprioceptive neuromuscular facilitation

Goals	Time allotment	Treatment protocol
Patient education	-	The patient was educated about his condition, advantages of receiving physiotherapy, and demonstration of breathing and ROM exercises, and proper counseling of the patient was done.
Increase mouth opening to one finger	Week 1	Practice opening and closing of mouth for 10 seconds. To enhance and sustain circulation, simple passive movements of the upper and lower limbs were taught.
To improve the ROM of TMJ	Week 2	Exercises like 4x4 (four exercises, four times), which increased functional jaw opening and controlled ROM, helped enhance the ROM of the TMJ. The secretion from both sides of the lower lobes of the lungs was removed using manual chest vibration and chest percussion.
To increase joint approximation and ROM in lower limbs	Weeks 4- 6	In 4-6 weeks of the treatment, mouth opening was improved. PNF was performed once or twice a week. D1 flexion and extension pattern of the PNF technique was used to improve the ROM (Figure 3). The stretching technique increased the ROM also.

Follow-up and outcome measures

The patient was able to complete all daily living activities without any difficulty, with a total score of 75/100 post-operatively and 45/100 pre-operatively according to the World Health Organization Quality of Life instrument (WHOQOL-bref). Additionally, the mouth opening was expanded to three fingers following physical therapy. The patient was ready and motivated to take part in the physical therapy. A 6/10 NPRS rating was given for the second postoperative day. After six weeks of rehabilitation post-surgery, improvements in NPRS were noted to be 2/10. The patient's shoulder and mouth opening ranges increased, indicating progress.

## Discussion

A study conducted by Mangulkar et al. discussed many therapeutic methods to place patients in informative settings, which included exercises for neck flexibility, breathing practices, mouth opening movements, and exercises including assistive strategies and mouth PNF. The most common postsurgical disadvantage that patients with oral cavity cancer experience, which makes daily tasks challenging, is restricted mouth opening. Patients with restricted mouth opening are treated effectively by physiotherapy using a variety of techniques, including goldfish exercises and mouth opening exercises, which improve patients’ condition and help them perform fundamental everyday tasks. Numerous stretching methods have been shown to provide therapeutic benefits.

A study conducted by Carroll et al. suggested that nine patients completed swallowing exercises before cardiac resynchronization therapy (CRT) and nine patients underwent mouth opening exercises following CRT as part of regular medical practice. Three months after the conclusion of the treatment, routine video fluoroscopy examinations were performed [9]. In the study conducted by Chen et al., exercises to strengthen the scapula were paired with motor control techniques for the voluntary motor group muscle. When executing a maximum voluntary isometric contraction, there is shoulder discomfort, active ROM of the shoulder abduction, and scapular muscular activity, which includes the serratus anterior muscles, middle trapezius, upper trapezius, and lower trapezius [10]. As suggested by Pyszora et al., the patient's overall health was enhanced by the physiotherapy program, which also lessened the severity of concomitant symptoms such as pain, tiredness, appetite loss, and depression [19]. Patients with advanced cancer receiving palliative care benefited from the physiotherapy program, which included physical exercises, myofascial release, and PNF treatments, in terms of cancer-related tiredness and other symptoms. Low molecular weight heparin for cancer patients with established venous thromboembolism (VTE) is the recommended medication for both initial and ongoing therapies [20].

## Conclusions

This case involved a 43-year-old male who had undergone anterolateral thigh buccal flap surgery. The patient quickly recovered, and mouth opening increased after a physical treatment that included mouth opening exercises. Therefore, patients with oral squamous cell carcinoma could benefit from these exercises. When mouth opening is restricted, it is challenging to keep the airway open, and the risk of aspiration is greatly enhanced. Additionally, percussion and vibration on both sides of the chest are given to remove secretion from the lungs.
